# Proposal for fasting insulin and HOMA-IR reference intervals based on an extensive Brazilian laboratory database

**DOI:** 10.20945/2359-4292-2023-0483

**Published:** 2024-10-17

**Authors:** Yolanda Schrank, Rosita Fontes, Andrea Faria Dutra Fragoso Perozo, Paula Bruna Araújo, Maria Fernanda Miguens Castelar Pinheiro, Dalva Margareth Valente Gomes, Luisane Maria Falci Vieira

**Affiliations:** 1 Diagnósticos da América S.A. São Paulo SP Brasil Diagnósticos da América S.A. (DASA), São Paulo, SP, Brasil; 2 Hospital Federal de Bonsucesso Rio de Janeiro RJ Brasil Hospital Federal de Bonsucesso, Rio de Janeiro RJ, Brasil; 3 Instituto Estadual de Diabetes e Endocrinologia Luiz Capriglione Rio de Janeiro RJ Brasil Instituto Estadual de Diabetes e Endocrinologia Luiz Capriglione (IEDE), Rio de Janeiro, RJ, Brasil; 4 Rio de Janeiro RJ Brasil Independent Researcher, Rio de Janeiro, RJ, Brasil; 5 International Federation of Clinical Chemistry and Laboratory Medicine Task Force Global Reference Intervals Database Milano Italy International Federation of Clinical Chemistry and Laboratory Medicine Task Force Global Reference Intervals Database, Milano, Italy

**Keywords:** Insulin, HOMA-IR, reference interval

## Abstract

**Objective:**

Fasting insulin and the homeostasis model assessment of insulin resistance (HOMA-IR) index are relatively simple and reliable noninvasive markers of insulin resistance (IR). Given the relevance of correctly diagnosing IR, we emphasize the importance of establishing reliable reference intervals (RIs) for these markers. This study aimed to determine the RIs of fasting insulin and HOMA-IR index in adults living in Rio de Janeiro and, secondarily, to verify potential RI differences between sexes.

**Subjects and methods:**

Serum insulin levels of 146,497 individuals (ages 20-60 years) who underwent blood sampling in the state of Rio de Janeiro were obtained retrospectively through access to an extensive laboratory database. Insulin was measured using the electrochemiluminescence immunoassay method. After applying exclusion criteria, 21,684 individuals (18,576 [86%] women) were included. The RIs were estimated using a computational mining approach that integrates a selection of R packages.

**Results:**

The 95% RIs in women and men and in the overall population were, respectively, 2.54-13.30 μU/mL (15.3-80.12 pmol/L), 2.43-11.89 μU/mL (14.6-71.7 pmol/L), and 2.52-13.14 μU/mL (15.2-79.2 pmol/L) for fasting insulin levels and 0.39-2.86, 0.38-2.81, and 0.39-2.86 for HOMA-IR values. Despite significant differences in insulin levels and HOMA-IR index between men and women, the use of sex-specific RIs was not justified.

**Conclusion:**

The RIs of fasting insulin levels and HOMA-IR values found in the overall population can be applied to both sexes. Thus, for our population, we suggest the RIs of 2.52-13.14 μU/mL (15.1-78.8 pmol/L) for fasting insulin and 0.39-2.86 for the HOMA-IR index.

## INTRODUCTION

Insulin resistance (IR) is an independent risk factor for the development of metabolic syndrome (MS), type 2 diabetes, hypertension, and coronary heart disease, the latter being a leading cause of morbidity and mortality. Other highly prevalent entities associated with IR are nonalcoholic fatty liver disease, polycystic ovary syndrome, hyperuricemia, and chronic renal disease (1,2). Predisposition to IR is multifactorial and has strong genetic and environmental influences (3). Many studies have shown that IR may predate the onset of type 2 diabetes by 10-20 years (4). Therefore, an early and proper diagnosis of IR is essential.

Dynamic tests – such as the hyperinsulinemic-euglycemic clamp – are considered reference techniques in diagnosing IR. However, these methods are invasive, expensive, time-consuming, and require experienced personnel, which limits their clinical utility (5,6). On the other hand, surrogate indices of IR, such as the homeostasis model assessment of insulin resistance (HOMA-IR) index and the quantitative insulin sensitivity check index (QUICKI), calculated from simultaneous fasting glucose and insulin levels, and even fasting insulin concentration, are simple, inexpensive, and validated alternative tools to assess IR (5,7). Some authors, including McAuley and cols., have observed better performance of fasting insulin compared with other noninvasive markers in predicting IR in the normoglycemic population (8).

Early assessment of fasting insulin and HOMA-IR through robust laboratory assays and reliable reference intervals (RIs) representative of each population (9,10) is critical for clinicians to identify individuals with IR. This, in turn, helps in recognizing those at risk of several highly prevalent comorbidities, particularly MS, type 2 diabetes, cardiovascular disease, and fatty liver disease.

Estimating and/or verifying RIs of quantitative clinical laboratory tests is essential to support the diagnosis, treatment, and monitoring of patients. The Clinical Laboratory Standards Institute (CLSI) EP28-A3c recommends clinical laboratories to establish RIs appropriate to the served population using "direct" methods. However, many problems are associated with these "conventional" approaches to establishing RIs. Recruiting a valid group by selecting a sufficiently large (usually > 120 individuals) and homogenous group of healthy reference individuals (where defining "health" becomes the initial problem) of various age groups and obtaining informed consent in today's environment is costly, time-intensive, and virtually an impossible task for most laboratories. In practice, very few laboratories perform their own RI studies, and most of them currently use RIs provided by reagent manufacturers. Owing to differences among individuals (*e.g.*, sex, age, weight, race, and geographic location) and analytical systems, the RIs provided by reagent manufacturers may not apply to the served population. In addition, laboratories and manufacturers often use references from studies conducted decades ago, despite substantial differences in methods and populations compared with current standards (11). An exciting, less labor-intensive, and increasingly applied alternative approach for establishing RIs is to perform an "indirect" *a posteriori* study of patient data already collected and stored in the laboratory database. The indirect approach assumes that most test results obtained during routine patient care are physiological and can, therefore, be used to derive RIs. To accomplish this, the proportion of physiological samples in the mixed input dataset is identified using different sophisticated statistical methods. Several publications have discussed this approach, and most of them were able to report clinically relevant and meaningful RIs. Statistically, it is more robust to analyze thousands of measurements that may include some unhealthy individuals than only 120 measurements that are assumed to be from healthy individuals (9).

With the advancement of information technology in recent years, the use of big data mining algorithms to establish RIs has gained increasing attention. These algorithms use real-world large-scale data from a clinical laboratory to distinguish the distribution of healthy individuals from a mixed distribution using graphs, iterations, parameter searches, and so on. They have the advantages of being low-cost, convenient, and time-saving to implement (12). Several data mining algorithms are currently used to establish RIs, including "Hoffmann's" method (13), "Bhattacharya's" method (14), Expectation-Maximization (EM) algorithm (15), "Kosmic" (16), and "refineR" model (17). More recently, a Brazilian mining model named Laboratory Reference Interval (LabRI) was compared with the "Kosmic" and "refineR" models and validated for the same purpose (18,19). In the meantime, numerous studies have established RIs for adult biomarkers using these algorithms (20–22).

Given the advances in the methodologies applied to determine RIs discussed above, and the significant heterogeneity of results currently available in the literature (23–27), this study aimed to determine the RIs of fasting insulin levels (using the Roche-Cobas platform) and HOMA-IR values in adults living in Rio de Janeiro, Brazil, and, secondarily, to verify potential RI differences between sexes.

## SUBJECTS AND METHODS

This cross-sectional, retrospective study analyzed information from individuals aged 20-60 years who underwent simultaneous laboratory evaluation of insulin, glucose, hemoglobin A1C (A1C), high-density lipoprotein cholesterol (HDL-c), and triglycerides (Tgs) from January to December 2019 in the state of Rio de Janeiro, obtained from an extensive database of a Brazilian Laboratory Information System (LIS). The spreadsheet generated initially contained 146,497 individuals, of whom 70% (102,380) were women.

To allow the exclusion of the main conditions potentially increasing the risk of elevated insulin – *e.g.*, obesity, diabetes, MS, hypertension, and dyslipidemia – the professionals who operate the LIS were requested to generate a spreadsheet with mandatory inclusion of the following information: sex, age, height, weight, insulin, glucose, A1C, HDL-c, Tgs, and medications in use. Some information contained in the spreadsheet, such as weight, height, medications in use, and duration of fasting, was obtained by receptionists checking in the patients at the laboratory. These data are collected routinely in the laboratory and are required to complete each patient's registration.

The exclusion criteria were body mass index (BMI) < 18.5 kg/m^2^ or ≥ 25 kg/m^2^, glucose level ≥ 100 mg/dL (5.55 mmol/L), A1C ≥ 5.7%, Tg level ≥ 150 mg/dL (1.69 mmol/L), HDL-c level < 50 mg/dL (1.3 mmol/L) for women and < 40 mg/dL (1.0 mmol/L) for men, and use of any of the following medications: oral or injectable antidiabetic agents, lipid-lowering medications, and anti-hypertensive drugs. To further reduce the possibility of including unhealthy individuals, those who underwent more than two yearly exams were excluded.

A total of 21,684 healthy individuals were included in the study after the application of the exclusion criteria and the exclusion of outliers (who could be individuals with unknown medical problems, leading to RI widening).

All biochemical tests were performed in the same certified Brazilian laboratory. Fasting for at least 8 hours was required. Notably, in the laboratories participating in the present study, patients are required to fast for at least 8 hours to undergo measurement of fasting glucose and insulin levels. Patients who do not comply with this requirement are rejected for blood sampling.

Fasting serum insulin was determined by the electrochemiluminescence immunoassay (ECLIA) method, using Roche Diagnostics kits and the Roche/Hitachi Cobas e-411 analyzer (Roche Diagnostics GmbH, Mannheim, Germany). A lyophilized quality-control material (Lyphochek Immunoassay Plus Control, Bio-Rad Laboratories, Hercules, CA, USA) was used to monitor the accuracy of the assays; the intra-assay and interassay coefficients of variation (CVs) were 1.2% and 3.5%, respectively. The insulin results were obtained in mU/L and converted to pmol/L (SI unit) using the conversion factor recommended by the World Health Organization (WHO) and the American Diabetes Association (1 μU/mL = 6.00 pmol/L) (28). Plasma glucose levels were measured by the hexokinase method using ADVIA 2400 Clinical Chemistry System (Siemens Healthineers, Erlangen, Germany). Levels of Tgs and HDL-c were analyzed using the enzymatic colorimetric method on a Modular Analyzer (Roche, Indianapolis, USA). Intra-assay and interassay CVs were both less than 2.0% for glucose, 1.8% for Tg, and 2.9% for HDL-c. Levels of A1C were determined by competitive turbidimetric inhibition immunoassay (TINIA) using Roche Diagnostics kits and the Roche/Cobas c 513 analyzer (Roche Diagnostics), an International Federation of Clinical Chemistry (IFCC) and National Glycohemoglobin Standardization Program (NGSP) certified A1C testing method.

The HOMA-IR index was calculated from individual fasting glucose and insulin concentrations using the following equation proposed by Matthews and cols. (29):


Fasting glucose(mmol/L)x fasting insulin(μU/L)/22.5

### Statistical analysis

After applying the exclusion criteria mentioned above, we proceeded to the indirect estimation of RIs by employing a computational approach that integrates a selection of R packages for optimization and accuracy. The automated pipeline includes algorithms for pre-processing data (removing outliers and statistical transformation after skewness and kurtosis analysis) for subsequent RI estimation. The univOutl package (30) played a crucial role in detecting and managing outliers. The MixR package (31) was employed for mixture deconvolution, and Modeest (32) and multimode (33) aided in the identification and analysis of modes and antimodes. Truncation points were determined based on a combination of information provided by the mixfit function of the MixR package. For RI estimation, the nonparametric percentile method was used in the truncated distribution. The RIs were determined as the central 95% insulin and HOMA-IR values. The 90% confidence interval (CI) was derived using methods adapted from the articles by Haeckel and Wosniok (34,35) and Solberg (36).

Insulin levels and HOMA-IR values did not follow a normal distribution and are, thus, expressed as medians and ranges. The Mann-Whitney test was used to compare variables between men and women. P values < 0.05 were considered statistically significant.

## RESULTS

After applying the exclusion criteria and excluding outliers, 21,684 individuals (18,576 [86%] women) with a median age of 38 ± 10.6 years were included in the analysis to determine the RIs of insulin and HOMA-IR. Given the great miscegenation of our population, we did not separate the data by race.

There were statistically significant differences between men and women regarding all characteristics, *i.e.*, men were younger and had higher BMI, glucose, Tgs, and A1C levels while women had more elevated fasting insulin, HOMA-IR index, and HDL-c levels (Table 1).

**Table 1:** Characteristics of the healthy individuals included in the analysis to determine reference values for fasting insulin and HOMA-IR index^a^

	Women (n = 18,576)	Men (n = 3,108)	P value^b^
Age, years	38.1 ± 10.3 (20-60)	36.3 ± 11.5 (20-60)	<0.001
BMI, kg/m^2^	22.7 (18.5-24.9)	23.5 (18.5-24.9)	<0.001
Insulin, μU/mL/pmol/L	6.61/39.8 (1.0-14.5/6.0-87.3)	6.54/39.4 (1.1-14.5/6.6-87.3)	<0.06
Glucose, mg/dL/mmol/L	89/4.9 (70-99/3.9-5.5)	93 (70-99/3.9-5.5)	<0.001
HDL-c, mg/dL/mmol/L	62/1.6 (50-124/1.3-3.2)	49 / 1.3 (40-119/1.0-3.0)	<0.001
Hemoglobin A1C, %	5.3 (3.4-5.7)	5.4 (4.2-5.7)	<0.001
Tgs, mg/dL (mmol/L)	73/0.8 (13-149/0.1-1.7)	83/0.9 (22-149/0.2-1.7)	<0.001
HOMA-IR	1.31 (0.19-3.51)	1.26 (0.23-3.47)	<0.001

Abbreviations: BMI, body mass index; HDL-c, high-density lipoprotein cholesterol; HOMA-IR, homeostatic model assessment of insulin resistance; Tgs, triglycerides.

a Because of skewed distribution, the results are presented as medians.

b Obtained using the Mann-Whitney test. To convert insulin levels from pmol/L to μU/mL, the values are multiplied by 0.166 (WHO recommendation). To convert glucose levels from mmol/L to mg/dL, the values are multiplied by 18.02 (manufacturer recommendation). To convert HDL-C levels from mmol/L to mg/dL, the values are multiplied by 38.67 (manufacturer recommendation). To convert triglycerides from mmol/L to mg/dL, the values are multiplied by 88.5 (manufacturer recommendation).

Peak insulin values were observed between the ages of 20-29 years, with a second rising trend between the ages of 50-60 years. In contrast, glucose levels increased progressively with age, reaching a peak between 50 and 60 years. The peak glucose level coincided with both peaks of Tg level and BMI value (Table 2).

**Table 2:** Distribution of body mass index values and serum insulin, glucose, triglyceride, and high-density lipoprotein cholesterol levels according to age group^a^

Age, Years	n	BMI, kg/m^2^	Insulin,μU/mL/pmol/L	Glucose, mg/dL/mmol/L	Tgs, mg/dL/mmol/L	HDL-c, mg/dL/mmol/L
20-29	5.467	22.58	7.25/43.7	86/4.8	74/0.84	62/1.60
30-39	8.217	22.83	6.28/37.8	88/4.9	70/0.79	65/1.68
40-49	5.179	23.01	6.03/36.3	88/4.9	74/0.84	65/1.68
50-60	2.821	23.07	6.37/38.4	89/4.9	78/0.88	66/1.70

Abbreviations: BMI, body mass index; HDL-c, high-density lipoprotein cholesterol; Tgs, triglycerides.

a Because of skewed distribution, the results are presented as median values. To convert insulin levels from pmol/L to μU/mL, the values are multiplied by 0.166 (WHO recommendation). To convert glucose levels from mmol/L to mg/dL, the values are multiplied by 18.02 (manufacturer recommendation). To convert triglyceride levels from mmol/L to mg/dL, the values are multiplied by 88.5 (manufacturer recommendation). To convert HDL-C levels from mmol/L to mg/dL, the values are multiplied by 38.67 (manufacturer recommendation).

Overall, 95% RIs for fasting insulin levels in women, men, and the overall population were, respectively, 2.54-13.30 μU/mL (15.3-80.12 pmol/L), 2.43-11.89 μU/mL (14.6-71.7 pmol/L), and 2.52-13.14 μU/mL (15.2-79.2 pmol/L). The corresponding HOMA-IR index RIs were, respectively, 0.39-2.86, 0.38-2.81, and 0.39-2.86 (Table 3).

**Table 3:** Reference intervals for serum insulin levels in women, men, and the overall study population (ages 20-60 years)

	n	95% RIs, μU/mL (pmol/L)	Lower (90% CI), μU/mL (pmol/L)	Upper (90% CI), μU/mL (pmol/L)
Women	18,576	2.54-13.30 (15.3-80.1)	2.49-2.59 (15.0-15.6)	13.22-13.38 (79.6-80.6)
Men	3,108	2.43-11.89 (14.6-71.6)	2.32-2.54 (13.9-15.3)	11.71-12.07 (70.5-72.7)
Overall population	21,684	2.52-13.14 (15.2-79.2)	2.48-2.56 (14.9-15.4)	13.06-13.22 (78.7-79.6)

Abbreviations: CI, confidence interval; RIs, reference intervals.

## DISCUSSION

This study proposes reference values for serum fasting insulin concentrations and HOMA-IR index in healthy Brazilian individuals selected from an extensive database of an important laboratory serving the entire state of Rio de Janeiro. Notably, before applying the exclusion criteria, 70% of the individuals included in the analysis were women, which is likely explained by the fact that women in our country seek more medical care and, consequently, undergo more laboratory tests than men. After applying the exclusion criteria, the proportion of women in our cohort increased even more (86%), showing that proportionally more men had pathologies that were part of our exclusion criteria, such as type 2 diabetes, MS, hypertension, obesity, and dyslipidemia.

In the present study, the RIs for fasting insulin levels were 2.54-13.30 μU/mL (15.3-80.12 pmol/L) in women, 2.43-11.89 μU/mL (14.6-71.7 pmol/L) in men, and 2.52-13.14 μU/mL (15.2-79.2 pmol/L) in the entire analyzed population. In line with these results, Tohidi and cols. studied 309 "healthy" Iranian individuals with characteristics similar to those of our population (124 men and 185 women, ages 24-83 years, mean age 40 years) and used the same equipment manufacturer (Roche Diagnostics kits and the Roche/Hitachi Cobas e-411 analyzer), reporting RIs for serum insulin in non-menopausal women, men, and overall population of 2.34-11.98 μU/mL (14.0-71.9 pmol/L), 1.61-11.37 μU/mL (9.7-68.2 pmol/L), and 2.11-12.49 μU/mL (12.7-74.9 pmol/L), respectively (37). Similarly, Francois and cols., studying a non-overweight French population, reported RIs for serum insulin concentration of 2.34-11.25 μU/mL (14.0-67.5 pmol/L) in women (aged 33-55 years, n = 157) and 2.08-11.82 μU/mL (12.5-70.9 pmol/L) in men (aged 37-58 years, n = 162) (38). In contrast, Li and cols. recently reported an RI for serum insulin concentrations in 1,434 "healthy" Chinese men without diabetes (age range 20-69 years) of 1.57-16.32 μU/mL (9.42-97.9 pmol/L) (39). The higher upper limit of their RI compared with ours (16.32 μU/mL or 77.3 pmol/L) may be due to our more stringent criteria for selecting healthy individuals and/or ethnic and racial differences.

The mean insulin levels in our study were slightly higher in women than in men. This finding has also been reported in other studies, including one by Tohidi and cols., who also found higher mean serum insulin levels in women than men (37). The trend of higher insulin levels in women than men may be related to body composition, since men tend to have a proportionally higher lean mass/fat mass ratio than women.

In addition to an initial increase in insulin levels at the beginning of adulthood, probably related to the increase in insulin levels observed in late adolescence (40), we highlight a trend of increasing insulin, glucose, and Tg levels, as well as increasing BMI with aging. This finding is corroborated by epidemiological studies and is associated with decreased peripheral insulin sensitivity, probably due to the reduction in lean body mass, increased BMI, and physical inactivity with aging (37). However, among very elderly individuals (ages ≥ 80 years), these markers show a downward trend, probably due to nutritional factors such as malnutrition, which is common in this age group (data not shown) (41).

The significant difference in mean insulin levels between men and women does not justify the adoption of sex-specific reference values because the ratio of standard deviations was < 1.5 (0.05) and the calculated z value of our cohort was less than the criterion dictating partitioning of reference values (11).

In the present study, the HOMA-IR index RIs in women, men, and the overall analyzed population were, respectively, 0.39-2.86, 0.38-2.81, and 0.39-2.86. These cutoff values are relatively higher compared with those reported in other studies (23,37,42). This variation reflects differences in selection criteria for the reference cohort and the diverse ethnicity of the analyzed populations. On the other hand, the upper limit result of the HOMA-IR index found in our study (2.86) was similar to that reported by Geloneze and cols. (2.71), who considered the 90^th^ percentile (we considered the 95^th^ percentile) and also studied a normal glucose-tolerant, non-obese, Brazilian population (43). Applying the same percentile used by Geloneze and cols. to our population, we would obtain a HOMA-IR cutoff value of 2.63.

As with fasting insulin levels, it is also not justifiable to use sex-specific reference values for the HOMA-IR index since the ratio of standard deviations was < 1.5 (0.03), and the calculated z value was less than the criterion dictating partitioning of reference values (11).

Since, by definition, MS is associated with IR, our reference population consisted necessarily of individuals without MS. According to the National Cholesterol Education Program Adult Treatment Panel III (NCEP-ATP III), MS represents the combination of at least three of the five following variables: diabetes, hypertension, abdominal obesity, hypertriglyceridemia, and low HDL-c (44). We did not have access to information about the individuals’ abdominal circumference or blood pressure levels, so we ensured the exclusion of MS in our reference population by enforcing the absence of the other three criteria. Although we did not have access to abdominal circumference or blood pressure levels, we had access to their self-reported weight and height, as well as medications in use. Therefore, we were able to exclude individuals who were overweight or obese, and through access to the individuals’ anti-hypertensive drugs in use, we could also exclude most individuals with hypertension, thus minimizing the presence of conditions associated with IR.

A concern with indirect methods is that the RIs may be wider than they should be or skewed due to the inclusion of potentially unhealthy individuals. This concern does not apply to the present study, as we observed RIs narrower than those of most manufacturers, probably due to the large number of individuals included in the analysis (27). As discussed above, the RIs obtained in our study coincide with those from other studies but are quite different from RIs recommended by manufacturers of commercially available test kits, which even vary widely among themselves (45–47).

Finally, a potential bias in the present study was a possible inconsistency between self-reported weight and height and actual measured values. However, studies have shown that differences between self-reported and measured weight and height usually have a negligible impact on BMI values. Carvalho evaluated 299 individuals living in São Paulo (112 men and 187 women) and observed that differences between reported and measured weight and height were not significant for either sex or age groups (48). Another potential bias was the self-reported use of medications, which may have been incomplete or incorrect in some cases, increasing the risk of including potentially unhealthy individuals in the study and, thus, resulting in falsely higher RIs for insulin and HOMA-IR. However, considering the large number of individuals included in the present study, we believe this potential bias was also minimized.

In conclusion, since the analysis of our data showed no clinically significant differences in sex-specific RIs for fasting insulin and HOMA-IR index, we conclude that separate RIs for men and women are unnecessary. Therefore, the RIs for fasting insulin and HOMA-IR index found in the overall analyzed population can be applied to both sexes. Based on that, we suggest the following RIs for our population: fasting insulin, 2.52-13.14 μU/mL (15.2-79.2 pmol/L); HOMA-IR index, 0.39-2.86.

We emphasize that despite the application of an indirect method to establish RIs, the large number of patients included in our study, the strict exclusion of possible factors that could influence fasting insulin, and the application of a robust method for insulin measurement make our results highly representative of the studied population.

## Data Availability

the data are stored in a database of the Brazilian Laboratory Information System (LIS). The Analytics team is responsible for handling the data, which are available upon request.
